# Antifibrotic Effect of Marine Ovothiol in an *In Vivo* Model of Liver Fibrosis

**DOI:** 10.1155/2018/5045734

**Published:** 2018-12-17

**Authors:** Mariarita Brancaccio, Giuseppe D'Argenio, Vincenzo Lembo, Anna Palumbo, Immacolata Castellano

**Affiliations:** ^1^Department of Biology and Evolution of Marine Organisms, Stazione Zoologica Anton Dohrn, Naples, Italy; ^2^Gastroenterology Unit, Department of Clinical Medicine and Surgery, School of Medicine, Federico II University, Naples, Italy; ^3^IBAF Institute, National Research Council (CNR), via P. Castellino, Naples, Italy

## Abstract

Liver fibrosis is a complex process caused by chronic hepatic injury, which leads to an excessive increase in extracellular matrix protein accumulation and fibrogenesis. Several natural products, including sulfur-containing compounds, have been investigated for their antifibrotic effects; however, the molecular mechanisms underpinning their action are partially still obscure. In this study, we have investigated for the first time the effect of ovothiol A, *π*-methyl-5-thiohistidine, isolated from sea urchin eggs on an *in vivo* murine model of liver fibrosis. Mice were intraperitoneally injected with carbon tetrachloride (CCl_4_) to induce liver fibrosis and treated with ovothiol A at the dose of 50 mg/kg 3 times a week for 2 months. Treatment with ovothiol A caused a significant reduction of collagen fibers as observed by histopathological changes and serum parameters compared to mice treated with control solution. This antifibrotic effect was associated to the decrease of fibrogenic markers involved in liver fibrosis progression, such as the transforming growth factor (TGF-*β*), the *α*-smooth muscle actin (α-SMA), and the tissue metalloproteinases inhibitor (TIMP-1). Finally, we provided evidence that the attenuation of liver fibrosis by ovothiol A treatment can be regulated by the expression and activity of the membrane-bound *γ*-glutamyl-transpeptidase (GGT), which is a key player in maintaining intracellular redox homoeostasis. Overall, these findings indicate that ovothiol A has significant antifibrotic properties and can be considered as a new marine drug or dietary supplement in potential therapeutic strategies for the treatment of liver fibrosis.

## 1. Introduction

In the last decades, the need to face complex challenges, i.e., the supply of sustainable food, human health, and aging population has stimulated research efforts to discover new active compounds from natural sources. In particular, the need to develop more efficient products and benefits for mankind, in order to treat or prevent many human disorders and to overcome the side effects of most of the approved drugs, has stimulated a great interest from pharmaceutical and nutraceutical industrial field to search for new natural products from less explored sources. In the last years, growing attention has been focused on the ocean characterized by a higher biodiversity compared to the earth [1]. In particular, marine natural products have inspired several approved pharmaceutical products, which are now in clinical use and/or in various stages of clinical development [2].

Liver fibrosis represents a worldwide health problem for its growing incidence and prevalence, and its evolution towards cirrhosis, which is associated with high morbidity and mortality. Thus, there is an urgent need to develop antifibrotic treatments that can prevent, halt, or even reverse liver fibrosis or cirrhosis [3]. Liver fibrosis results from chronic liver injury during a long-term wound-healing response, which causes increasing excessive accumulation of extracellular matrix (ECM) proteins, leading to fibrogenesis and later cirrhosis [4]. It represents a complex process that includes apoptosis of hepatocytes, infiltration of inflammatory cells, induction of inflammatory cytokines, and proliferation of nonparenchymal cells producing ECM, mainly hepatic stellate cells (HSCs) [5]. Active HSCs are characterized by increased proliferation, migration, and contractility, and a relative resistance to apoptosis. At the molecular level, they show increased expression of *α*-smooth muscle actin (*α*-SMA) and procollagen-I; both associated with the ability of the activated HSCs to depose collagens and other matrix proteins in the extracellular space [6]. Indeed, activated HSCs present an altered regulation of matrix remodeling enzymes, such as metalloproteinases (MMPs) and their tissue inhibitors (TIMPs), modulating matrix degradation and production, respectively [7]. Another key player in fibrosis development is the pleiotropic cytokine TGF-*β*1, which is secreted in the latent form and when active, induces the activation of HSCs and modulates the expression and secretion of a number of proteases and their regulators, including MMPs and TIMPs [8]. TGF-*β*1 can also auto-induce its own production thus subsequently amplifying its actions [9, 10].

Moreover, liver functionality is finely regulated by glutathione (GSH) levels, the most abundant cellular thiol in the cells. GSH is synthesized inside the cell and partially secreted in the extracellular space along a concentration gradient. In the extracellular space, GSH is hydrolyzed by *γ*-glutamyl-transpeptidase (GGT), a dimeric enzyme located on the membrane surface, and highly expressed in the liver and kidney [11]. This enzyme is therefore involved in GSH metabolism, amino acids recycling, and detoxification mechanisms. In detail, it catalyzes the hydrolysis of the *γ*-glutamyl bond in GSH and the transfer of the *γ*-glutamyl group to amino acids and small peptides. It often catalyzes the *γ*-glutamylation of the administered drugs, allowing the liver and the kidney to detoxify the organism [11, 12].

Several studies demonstrated the efficacy of different natural products and phytochemicals present in foods and used as food extracts (such as sulforaphane, S-allylcysteine, curcumin, proanthocyanidins, garlic extract, coffee, and grape skin or seeds) to prevent or reduce liver fibrosis progression by different mechanisms in several animal models [10, 13–17]. However, despite the striking progress in understanding the molecular mechanisms involved in liver fibrosis and cirrhosis, the antifibrotic therapies are still lacking. In this context, methyl-5-thiohistidines, also called ovothiols, isolated in huge amounts from the eggs of marine invertebrate species, represent promising bioactive compounds [18–20]. First of all, they display unusual antioxidant properties due to the peculiar position of the thiol group on the imidazole ring of histidine [21, 22]. Thanks to their chemical properties, ovothiols can recycle oxidized GSH and play a key role in controlling the cellular redox balance [23, 24]. These molecules were found in different methylated forms at the amino group of the lateral chain of histidine in several marine invertebrates [19, 20, 25–27]. For example, ovothiol A, unmethylated on the lateral chain, was isolated from the eggs of the sea urchin *Paracentrotus lividus* [25] and the sea cucumber *Holothuria tubulosa* [26]. Ovothiol B, mono-methylated on the lateral chain of histidine, was found in the ovaries of the scallop *Chlamys hastata* [21]. Ovothiol A has also been recently identified in the green microalga *Euglena gracilis*, which is a rich source of vitamins and antioxidants [28]. Interestingly, the biosynthetic pathway leading to ovothiols lacks in vertebrates [18]; therefore, mammals do not produce autonomously these molecules. Up to date, a very few studies have focused on the biological activities of ovothiols in human model systems. Several years ago, a synthetic analogue of ovothiol was reported as a neuroprotective agent in a murine model of brain injury [29]. Recently, we have claimed the nutraceutical and/or pharmaceutical use of marine ovothiol to relieve pathologies associated with chronic endothelial dysfunction, such as diabetes [30], and suggested a role of the molecule in regulating cell proliferation in human liver tumor cells [31]. In particular, ovothiol A, purified by sea urchin eggs, was shown to induce autophagy in human hepatocarcinoma cell lines.

The aim of this study was to evaluate in depth the effect of ovothiol A in an *in vivo* model of chronic liver inflammation, which can silently progress, leading to cirrhosis and eventually to hepatocarcinoma, which is one of the most aggressive tumors with a very poor prognosis. We used a murine model of liver fibrosis in order to test if the administration of ovothiol A in its disulfide form could induce the recovery of liver functionality. In order to define the molecular mechanism underpinning this process, we tested the gene and protein expression of different fibrogenic markers (*α*-SMA, TGF-*β*1, and TIMP1) and the activity of the mature membrane-bound GGT form. Overall, our experiments point to evaluate the efficacy of ovothiol A as a novel anti-inflammatory and antifibrotic bioactive molecule.

## 2. Methods

### 2.1. Experimental Model of Progressive Fibrosis and Animal Treatment

Male balb-c albino mice (20–25 g) were housed in a room at a mean constant temperature of 22°C with a 12 h light–dark cycle and free access to standard pellet chow and water. The study was approved by Federico II University School of Veterinary Medicine Animal Care N° 104/2015-PR. Liver fibrosis was induced in mice by intraperitoneal (ip) injection of carbon tetrachloride (CCl_4_) 0.2 mL/100 g body weight (b.w.) in refined olive oil (1 : 1) twice a week for 7 weeks according to a well-established protocol [32]. Two experimental groups were designed as follows: (1) mice receiving CCl_4_ and ip injection of the disulfide form of ovothiol A 50 *μ*g/g b.w. 3 times a week for 7 weeks (*n* = 7); (2) control group receiving CCl_4_ and ip injection of vehicle (aqueous solution) alone (*n* = 7) with the same timing. Ovothiol A was prepared as described by Russo et al., 2014 [31]. The dose of administration was chosen on the basis of a previous work demonstrating that at this posology an ovothiol analogue induced no toxicity in not injured mice and neuroprotection in mice affected by brain injury [29]. A group of 5 normal mice was also included in the study. The animals were then killed under anaesthesia, and their livers were harvested at peak fibrosis (3 days after the final injection of CCl_4_). After harvesting, livers were divided with a minimum of two lobes fixed in formalin for histologic analysis and histochemistry, and the remaining tissue was snap-frozen for RNA and protein extraction. Serum was also collected from each mouse to analyze biochemical parameters.

### 2.2. Histology and Determination of Serum Biochemical Parameters

Formalin-fixed paraffin-embedded tissue was cut into 4 *μ*m sections by using routine techniques and mounted onto slides with coverslips. Representative sections of each fixed liver were stained with haematoxylin/eosin (H&E) for routinely observations. For the detection of collagen content, sections were stained with Sirius red/Fast green, according to standard protocols. All histological analyses were performed by an experienced histopathologist in a blinded manner. For each mouse, 64 fields of a constant raster of 31 mm^2^ were analyzed at 100-fold final magnification. For semiautomated morphometry, a Sony 3CCD (model DXC-950P) video microscope equipped with a motor stage and the Quantimed 500MC (Leica, Germany) software were used. Serum aspartate aminotransferase (AST), alanine aminotransferase (ALT), and alkaline phosphatase (ALP) levels were determined to assess liver function by using standard laboratory techniques and equipment (Roche Diagnostics, Germany).

### 2.3. RNA Extraction and cDNA Synthesis

Total RNA was extracted from frozen tissue by homogenization in Trizol Reagent according to the manufacturer's protocol (Life Technologies). The amount of total RNA extracted was estimated measuring the absorbance at 260 nm and the purity by 260/280 and 260/230 nm ratios by Nanodrop (ND-1000 UV-Vis Spectrophotometer; NanoDrop Technologies). The integrity of RNA was evaluated by agarose gel electrophoresis. For each sample, 1200 ng of total RNA extracted was retro-transcribed with iScript^TM^ cDNA synthesis kit (Bio-Rad), following the manufacturer's instructions.

### 2.4. Gene Expression by Real-Time qPCR

For real-time qPCR experiments, the data from each cDNA sample were normalized using the mouse housekeeping gene GAPDH (glyceraldehyde 3-phosphate dehydrogenase). In the case of GAPDH, TGF-*β*, *α*-SMA, Col1a1, and GGT-1, the specific primers were designed based on the nucleotide sequences downloaded by NCBI database (accession numbers) using Primer3WEB v.4.0.0. GAPDH primer forward 5′-GGTGAAGGTCGGTGTGAACG-3′, primer reverse 5′- CTCGCTCCTGGAAGATGGTG-3′; TGF-*β* primer forward 5′- TGCGCTTGCAGAGATTAAAA-3′, primer reverse 5′-CTGCCGTACAACTCCAGTGA-3′; *α*-SMA primer forward 5′-CTGACAGAGGCACCACTGAA-3′, primer reverse 5′-CATCTCCAGAGTCCAGCACA-3′; Col1a1 primer forward 5′-ACAGTCGCTTCACCTACAGC-3′, primer reverse 5′-TGGGGTGGAGGGAG;TTTACA-3′ GGT-1 primer forward 5′-TGCTCGGTGACCCAAAGTTT-3′, primer reverse 5′-TTCAGAGGATGGCAGTGCTG-3′. A final concentration of 1.4 pmol/*μ*L for each primer and 1 FastStart SYBR Green Master Mix (total volume of 10 *μ*L) were used for the reaction mix. PCR amplifications were performed in a ViiA^TM^ 7 real-time PCR system (Applied Biosystems) thermal cycler using the following thermal profile: 95°C for 10 min, one cycle for cDNA denaturation; 95°C for 15 sec and 60°C for 1 min, 40 cycles for amplification; 72°C for 5 min, one cycle for final elongation; and one cycle for melting curve analysis (from 60°C to 95°C) to verify the presence of a single product. Each assay included a no-template control for each primer pair. Specificity of amplification reactions was verified by melting curve analysis. The efficiency of each primer pair was calculated according to standard method curves using the equation E = 10^–1/slope^. Five serial dilutions were set up to determine Ct values and reaction efficiencies for all primer pairs. Standard curves were generated for each oligonucleotide pair using the Ct values versus the logarithm of each dilution factor, and PCR amplifications were performed in a ViiA^TM^ 7 real-time PCR system (Applied Biosystems) thermal cycler using the standard protocol previously reported. To capture intra-assay variability, all real-time qPCR reactions were carried out in triplicate. Fluorescence was measured using ViiA^TM^ 7 Software (Applied Biosystems). The expression of the genes was analyzed and internally normalized against GAPDH using relative expression software tool (REST) software based on the Pfaffl method (2002). Relative expression ratios above two cycles were considered significant. Experiments were repeated at least three times.

### 2.5. Protein Analysis

Liver samples, about 20/30 mg, were homogenized in 1 mL of RIPA lysis buffer (1X) containing a 50 mM Tris-HCl (pH 7.6), 150 mM NaCl, 5 mM EDTA, 0.5% NP-40, 0.5% Sodium deoxycholate, 10% SDS, phosphatase, and protease inhibitor cocktail (Roche). Liver homogenates were run on 12% SDS/polyacrylamide gel according to Laemmli. Following electrophoresis, proteins were transferred onto a PVDF (Millipore) membrane (Bio-Rad Trans-Blot Apparatus) and detected using a mouse anti-TGF-*β* polyclonal antibody (Sigma-Aldrich, USA), mouse anti-GGT monoclonal antibody (Santa Cruz Biotechnology, USA), rabbit anti-*α*-SMA monoclonal antibody (Santa Cruz Biotechnology, USA), rabbit TIMP-1 polyclonal antibody (Elabscience), and as an internal control mouse anti-GAPDH monoclonal antibody (Santa Cruz Biotechnology, USA). All primary antibodies were incubated at 4°C overnight. The appropriate secondary antibody was added, and immunoreactive proteins were detected using the ECL (WesternBright^TM^ detection kit ECL, Advansta, USA) according to the manufacturer's instructions. Protein expression levels were analyzed by means of densitometric analysis using the Image J software.

### 2.6. Enzyme Isolation—GGT Activity

Tissues were homogenized with Potter-Elvehjem tissue homogenizer at 4°C in 5 volumes of 25 mM Tris-HCl, pH 7.5, 0.2 mM EDTA, containing 0.33 M sucrose, 1 *μ*M leupeptin, and 1.4 *μ*g/mL aprotinin [33]. The homogenate was centrifuged at 9000 × *g* for 20 min; the supernatant was spun at 100,000 × g for 1 h to spin down nuclei, mitochondria, and cellular debris. The pellet was homogenized in 25 mM Tris-HCl, pH 7.35, 0.5% Triton X-100, 1 *μ*M leupeptin, 1.4 *μ*g/mL aprotinin, and then centrifuged again at 100,000 × *g* for 1 h. The supernatant was aliquoted and stored at −80°C and then assayed for GGT protein expression and activity. GGT activity was determined by a colorimetric test. The assay buffer contains 100 mM Tris-HCl pH 7.8 or PBS 1X pH 7.4. Each reaction contains 1 mM of *γ*-glutamyl-para-nitroanilide as a donor substrate and 40 mM glycylglycine as an acceptor substrate. The product formation, p-nitroaniline, was continuously monitored at room temperature at A405 nm using a Bio-Rad 680 microplate reader with Microplate Manager 5.2 (Bio-Rad) software. One unit of GGT activity was defined as the amount of GGT that released 1 *μ*mol of p-nitroaniline/min at room temperature.

### 2.7. Glutathione Assay

Total glutathione levels were determined by Glutathione Assay Kit (Sigma). Briefly, frozen liver tissues were ground with a pestle with a mortar in the presence of liquid nitrogen to prepare a fine powder. Then, 100 mg of powder was added to 3 volumes of 5% 5-sulfosalicylic acid and mixed. Then, other 7 volumes of 5% 5-sulfosalicylic acid were added, mixed, left for 5 min at 4°C, and finally centrifuged at 10,000 × *g* for 10 min. Diluted samples of the supernatants were used for the assay procedure, in which following the incubation with glutathione reductase and NADPH, glutathione was totally recovered in the reduced form and thus determined by monitoring the reduction of 5,5-dithiobis (2-nitrobenzoic acid) to 5-thio-2-nitrobenzoic at 412 nm by a Thermo Scientific™ Multiskan™ FC Microplate Photometer.

### 2.8. Statistical Analysis

As appropriate, comparisons among groups were made by Student's *t*-test or analysis of variance ANOVA followed by Bonferroni or Tukey's multiple comparison tests. Values of *p* < 0.05 were considered significant.

## 3. Results

### 3.1. Effect of Ovothiol on Liver Histology and Serum Biochemical Parameters

**Figure 1:**
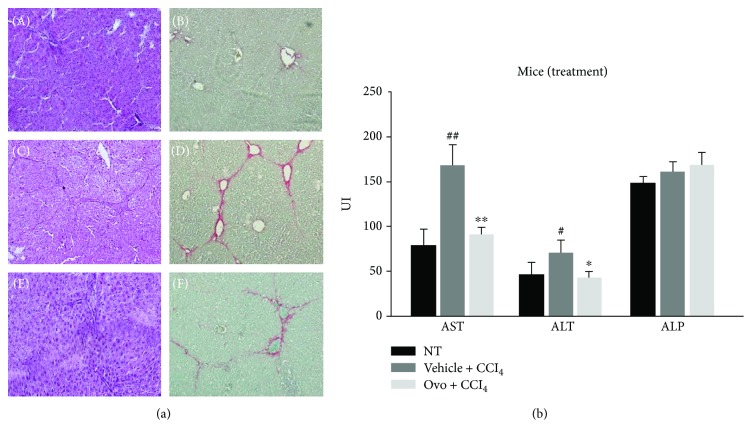
(a) Histological analysis. H&E staining and sirius red dye to highlight the collagen fibers of liver section: (A, B) healthy hepatic tissue; (C, D) fibrotic liver tissue induced by CCl_4_; (E, F) liver tissue with hepatic fibrosis treated with ovothiol A. (b) Evaluation of serum levels of liver enzymes. The levels of AST, ALT, and ALP were determined in the serum from mice affected by liver fibrosis and treated with ovothiol A or control solution. Data are expressed as mean ± SD, *n* = 7. The significance was determined by the ANOVA and post hoc analysis: (^∗^*p* < 0.05) and (^∗∗^*p* < 0.01) represent significance compared to vehicle + CCl_4_; (^#^*p* < 0.05) and (^##^*p* < 0.01) compared to nontreated (NT) healthy mice.

In order to evaluate the effect of ovothiol A on relieving induced liver fibrosis, the histology and functionality of the liver were examined in injured mice treated and not treated with ovothiol compared to control animals. Histological analysis of hepatic tissue of mice treated with CCl_4_ for 7 weeks compared with healthy mice tissue confirmed that the injection of CCl_4_ caused the progression of hepatic fibrosis, as demonstrated by the increase of red colored collagen fibers showing established septa linking hepatic veins (see Figure 1(a), C–D compared to A and B). Ovothiol A treatment significantly reduced hepatic fibrosis as shown by the reduction of red colored collagen fibers (Figure 1(a), E–F compared to C and D). The amount of collagen was reduced in fibrotic mice treated with ovothiol compared to those treated with vehicle alone (2.7 ± 0.9% vs 5.8 ± 1.2%, *p* < 0.05).

Serum levels of liver enzymes, aspartate aminotransferase (AST), alanine aminotransferase (ALT), and alkaline phosphatase (ALP) were assayed to evaluate liver functionality. Levels of AST and ALT increased in mice affected by hepatic fibrosis, whereas the treatment with ovothiol A led to a significant reduction in AST and ALT levels (Figure 1(b)). Conversely, serum ALP levels in mice with hepatic fibrosis did not significantly differ among the treatment groups.

### 3.2. Effect of Ovothiol on Gene Expression of Biomarkers of Liver Fibrosis

To evaluate the transcript regulation of specific liver fibrotic markers, gene expression analysis was carried out by real-time qPCR on TGF-*β*, *α*-SMA, GGT, and fibrillar type collagen 1 (Col1a1).

**Figure 2:**
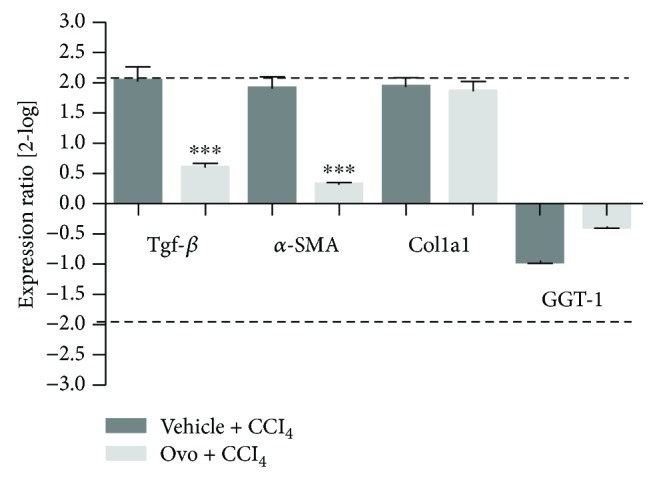
Gene expression analysis of markers of liver fibrosis by real-time qPCR. The levels of gene expression of the fibrotic markers in tissues after treatment with ovothiol A or control solution were compared to tissues from healthy mice (reference baseline). Data were analyzed through the REST software, which considers fold differences ≥ +/−2 to be significant. (^∗∗∗^*p* < 0.001) represents the significance compared to the treated mice with vehicle + CCl_4_.

A significant increase in gene expression for TGF-*β*, *α*-SMA, and Col1a1 was observed in mice affected by liver fibrosis and treated with control solution compared to healthy mice (Figure 2), whereas no significant variation was shown for GGT expression levels. On the other hand, samples from mice treated with ovothiol A showed a significant downregulation of mRNA of the TGF-*β* and *α*-SMA, whereas the gene expression of Col1a1 and GGT were not affected.

### 3.3. Effect of Ovothiol on Protein Expression of Key Players in Liver Fibrosis Progression

**Figure 3:**
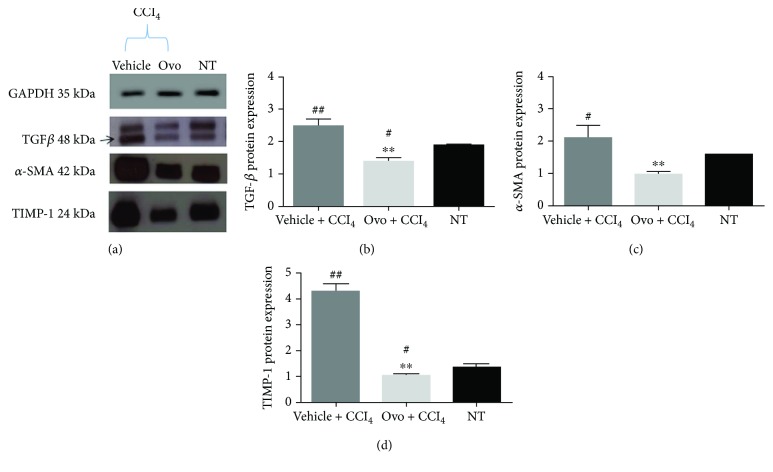
Protein expression of liver fibrosis markers. (a) A representative experiment of Western blot analysis of cytosolic extracts obtained from hepatic tissues of mice treated with ovothiol A or vehicle, after induction of liver fibrosis, compared to samples of healthy mice (NT), using antibodies specific for TGF-*β*, *α*-SMA, and TIMP1. Histograms of the densitometry analysis of protein bands obtained by Western blot for liver markers: (b) TGF-*β*; (c) *α*-SMA; and (d) TIMP1. Data were normalized for GAPDH. Data are expressed as mean ± SD, *n* = 7. The significance was determined by ANOVA test. (#*p* < 0.05) and (##*p* < 0.01) represent significance compared to NT; (^∗∗^*p* < 0.01) represents significance compared to the treated with vehicle + CCl_4_.

The protein expression of the hepatic fibrogenic markers, TGF-*β*, *α*-SMA, and TIMP1 was evaluated by Western blot analysis (Figure 3(a)). In mice affected by liver fibrosis, the levels of TGF-*β*, *α*-SMA, and TIMP1 significantly increased compared to healthy mice. After treatment with ovothiol A, the protein expression of TGF-*β*, *α*-SMA, and TIMP1 significantly decreased compared to mice with hepatic fibrosis (Figures 3(a)–3(d)).

**Figure 4:**
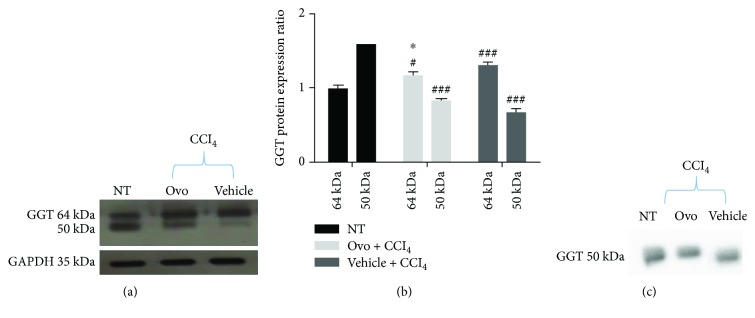
Protein expression of GGT. (a) Representative Western blot performed with GGT-specific antibody using microsomal extracts of hepatic tissues of mice with liver fibrosis treated with ovothiol or control solution compared to nontreated healthy mice (NT). (b) Histogram of the densitometric analysis of the GGT bands at 64 and 50 kDa. Data were normalized for GAPDH. (c) Representative Western blot performed on mice serum using GGT-specific antibody. Data are expressed as mean ± SD, *n* = 6. Statistical significance was determined by the one-way ANOVA test; (#*p* < 0.05) and (###*p* < 0.001) represent the significance compared to the corresponding NT band; (^∗^*p* < 0.05) represents the significance compared to fibrotic mice treated with control solution.

The presence of GGT in the liver tissue was also evaluated by immunoblot of microsomal extracts containing membrane-bound GGT. GGT is synthesized as a single 64 kDa precursor polypeptide, which undergoes self-proteolysis to form the mature protein composed of two subunits, the largest of which is around 50 kDa (Figure 4(a)). The antibody used in this study is directed against a peptide contained in the major subunit; therefore, it is able to recognize both the large subunit and the precursor. In the liver microsomal extracts of healthy mice, 62% of the total GGT is present in the mature form, while in the fibrotic tissues, the mature form significantly decreased to 36%. Conversely, the treatment with ovothiol A induced an increase in mature protein up to 44% of the total content of GGT (Figures 4(a) and 4(b)). The presence of GGT was also evaluated in mice serum. In all three groups of mice, the serum contained only one band recognized by GGT antibody corresponding to the size of the large subunit of the mature form (Figure 4(c)).

### 3.4. Ovothiol Affects Membrane-Bound GGT Activity

**Figure 5:**
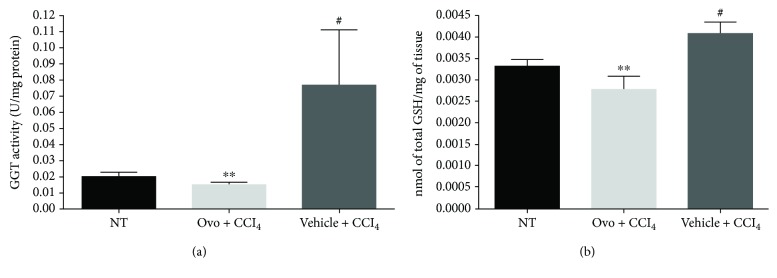
GGT activity and glutathione content. (a) The enzymatic activity of GGT was evaluated on liver tissue microsomal extracts containing membrane-bound GGT. The activity of GGT was normalized compared to the mature protein band (50 kDa) detected by Western blot. (b) The levels of glutathione were determined in hepatic tissue of mice treated with ovothiol A or vehicle, after induction of hepatic fibrosis, compared to samples of healthy tissue mice (NT). Data are expressed as mean ± SD, *n* = 6. The bars indicated the mean of 7 measures +/− SD (standard deviation). The significance was determined by the ANOVA and post hoc analysis: (#*p* < 0.05) represents significance compared to healthy control; (^∗∗^*p* < 0.01) represents significance compared to mice treated with vehicle + CCl_4_.

GGT activity was determined in liver microsomal extracts and normalized against the amount of the mature protein, which represents the active form of the enzyme, compared to the inactive precursor polypeptide [11, 12]. GGT activity significantly increased in fibrotic tissues compared to healthy mice (Figure 5(a)). Treatment with ovothiol A, on the other hand, induced a significant reduction in GGT activity. Since GGT activity is closely related to glutathione metabolism, we also evaluated total glutathione levels in the hepatic tissues. As shown in Figure 5(b), total glutathione levels increased in tissues from mice affected by liver fibrosis and treated with vehicle solution and decreased in liver tissues from mice treated with ovothiol A.

## 4. Discussion

In the last decade, studies on the isolation and structural characterization of ovothiols have attracted the attention of many scientists [19, 20]. These molecules are sulfur-containing compounds derived from histidine, which, despite the relative structural simplicity, are likely involved in different biological processes in marine organisms [19, 20]. Of particular interest is the finding that vertebrates lack the biosynthetic pathway leading to ovothiol [18], thus envisaging new perspectives on the possible pharmacological applications of the molecule in humans [20, 34]. On this basis, great efforts have been devoted to the chemical synthesis of this class of compounds, lately leading to ovothiols [35, 36] or to 5-thiohistidine, the precursor of ovothiols, unmethylated at the imidazole ring [37]. However, the described chemical procedures are somehow cumbersome and not environmentally friendly; thus, the need to develop an eco-sustainable production of the molecule [20].

Previous studies showed that a synthetic ovothiol analogue exhibited neuroprotective activity in an *in vivo* model of brain injury [29], whereas the natural molecule ovothiol A in disulfide form was tested only in *in vitro* models, showing anti-inflammatory activity in human endothelial cells [30] and antiproliferative effect on human hepatic carcinoma cell lines [31]. These pleiotropic behaviors have been ascribed to different mechanisms of action of these molecules in different cellular contexts [20].

In this study, to deepen the anti-inflammatory properties of this class of molecules, we have evaluated the effect of ovothiol A on an *in vivo* model of liver fibrosis, which is a condition common to many chronic liver diseases, characterized by the presence of parenchymal damage [4, 5]. The chronic activation of the tissue repair response which leads to liver fibrosis is characterized by a significant accumulation of ECM and numerous profibrogenic markers, which is responsible for the excessive deposition of collagen fibers [6]. Regeneration from liver fibrosis implies key processes, such as the eradication of pathological agents, apoptosis of HSC, remodeling of ECM, and regeneration of parenchyma and liver function. From the clinical perspective, fibrinolytic therapies reverting advanced fibrosis after the elimination of the causative agent represent a feasible challenge [3]. In fact, in clinical practice, the spontaneous recovery of liver histology, after the elimination of the agent causing chronic liver disease, usually occurs only in some patients and takes more than 3 years. Several sulfur-containing compounds have been shown to induce the reversion of liver fibrosis [10, 13, 14]. Recently, an ergothioneine-rich diet has been shown to ameliorate liver fibrosis [38]. Ergothioneine is a sulfur-containing histidine produced by some fungi and bacteria [39]. Unlike ovothiol, the sulfur of ergothioneine is localized on position 2 of the imidazole ring of histidine, making it stable in its thionic form. In our study, the methyl-5-thiohistidine (ovothiol A) isolated from sea urchin eggs was administered as disulfide because the reduced form is unstable and very reactive and can be presumably generated in the cell by recycling mechanisms [29]. In the disulfide form, ovothiol A inhibited the onset and/or progression of hepatic fibrosis, highlighting the antifibrotic properties for this type of molecules. Our findings clearly indicate that ovothiol A induced a significant reduction in the accumulation of collagen fibers in injured hepatic tissues associated with the decrease of the serum levels of the liver enzymes AST and ALT (Figure 1). Indeed, ovothiol A activates fibrinolytic processes on the extracellular ECM matrix through the downregulation of different fibrotic markers (Figure 2). In particular, TGF-*β*, the most important profibrogenic cytokine, was found significantly reduced by ovothiol A treatment also at the protein level (Figure 3). Since the activation of TGF-*β* is a fundamental step for the activation of HSC and for hepatic fibrogenesis, it is reasonable to infer that a decreased expression of TGF-*β*, mediated by ovothiol A, is consistent with a reduced amount of activated HSC, remodeling of extracellular matrix, and regeneration of liver function. The reduction of activated HSC after ovothiol A treatment was further confirmed by the reduction of the *α*-SMA protein isoform, which generally increases in acute hepatopathies, and it is rarely detected in the normal liver.

On the other hand, our results demonstrate that the administration of ovothiol A had no effects on the gene regulation of Col1a1 (Figure 2), a known marker of activation of the fibrogenic process, which encodes the alpha-1 subunit of fibrillar type collagen [6]. The deposition of this protein is responsible for the significant changes that occur in the composition of the ECM following liver injury and consequent alteration of the liver tissue anatomy. The reduction of collagen fibers observed at histological level is therefore likely caused by an indirect action exerted by ovothiol A on the metalloproteinases and on the inhibitors thereof, which are involved in the degradation of ECM and consequently in the reversion of the pathological condition. Indeed, we found an increased protein expression of the metalloproteinase inhibitor TIMP1 in fibrotic tissues and a decreased expression after treatment with ovothiol A (Figure 3). Under these conditions, in fact, metalloproteases should be free to degrade collagen fibers in the ECM, thus reducing the fibrotic process.

The analysis of protein expression and activity of GGT appeared to be more complex. Our finding indicates that liver fibrosis is characterized by high levels of membrane-bound GGT activity (Figure 5(a)). The total content of GGT protein, consisting of the precursor and the mature form remained unchanged following ovothiol treatment (Figures 4(a) and 4(b)), according to the absence of GGT gene regulation (Figure 2). However, the amount of the membrane-bound mature form decreased in mice affected by liver fibrosis compared to control mice and was restored following ovothiol treatment (Figures 4(a) and 4(b)). The main function of GGT is to regulate the intracellular redox homeostasis by catalyzing the degradation of extracellular GSH and promoting thiols recycling within the cell [11, 12]. The cysteinylglycine, resulting from glutathione hydrolysis, is one of the most reactive thiol compounds able to reduce oxygen by a redox reaction with the iron ion, thus promoting the increase in reactive oxygen species (ROS) and oxidative reactions [40, 41]. Therefore, high levels of GGT activity may induce oxidative stress in the cell, thus contributing to the damage and development of liver fibrosis. It is well known that chronic inflammation of the liver can cause damage to the membranes resulting in the release of GGT into the blood [42]. Indeed, GGT and in particular its high serum levels are well-known markers of liver damage pathologies. However, the role of GGT anchored to the outer membrane surface of liver cells is not yet clear. Here, we show that during the process of liver fibrosis, the activity of the mature GGT form anchored to the membrane increases (see Figure 4), probably contributing to oxidative stress and membrane damage, which in turn causes the further release of the mature protein in the serum (Figure 4(c)). Treatment with ovothiol A in mice affected by the fibrotic process causes the reversion of this phenomenon, that is, the reduction of membrane GGT activity at the physiological levels of healthy mice. Indeed, by inhibiting GGT activity, ovothiol A should also reduce the amount of cysteinylglycine in the cell, thus avoiding the accumulation of ROS and oxidative damage. In support of this hypothesis, intracellular thiol levels increased in untreated fibrotic tissues and decreased following ovothiol A treatment (Figure 5(b)). This may contribute to maintain membrane integrity and reduce the release of the mature membrane-bound GGT into the serum. Indeed, our data suggest that the mature form of GGT is mostly released in the blood during liver fibrosis and that the treatment with ovothiol A presumably reduces this release, resulting in new mature membrane-bound GGT (Figure 4(c)). Another possible explanation could be that the progression of liver fibrosis negatively affects the auto-catalytic cleavage of the GGT precursor, whereas ovothiol A treatment can reinduce the maturation process. The finding that only the mature form of GGT is released in the serum may depend on the adverse effect of its own overexpression and activity or to a more pronounced anchorage to the membrane of its precursor form. Further studies will be needed to clarify these aspects.

## 5. Conclusions

**Figure 6:**
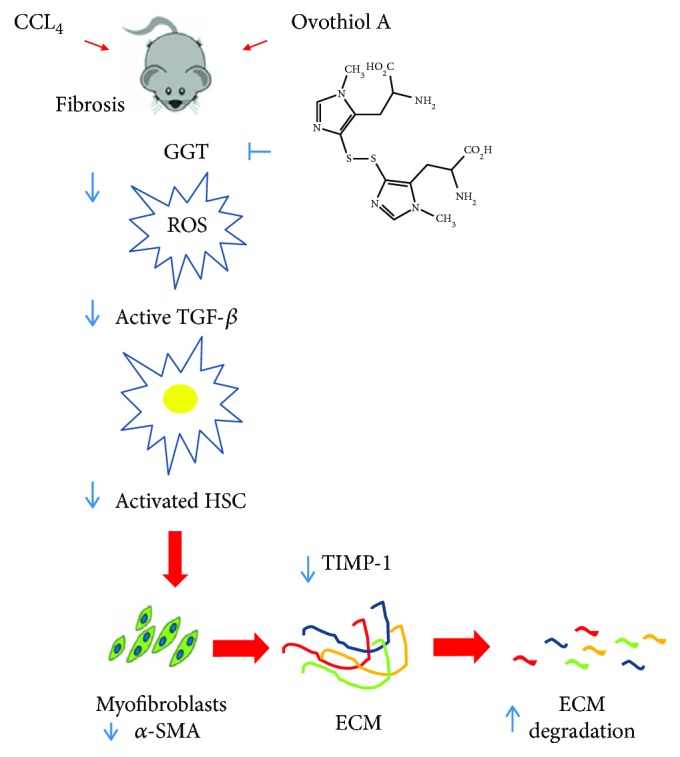
Proposed mechanism of action for ovothiol. During the development of liver fibrosis, membrane-bound GGT activity increases, leading to ROS overproduction. ROS can activate TGF-*β*, which in turn upregulates *α*-SMA and TIMP1, favoring ECM deposition. Ovothiol acts as a GGT inhibitor and in turn reduces TGF-*β* activation, thus inducing a cascade of events leading to downregulation of profibrogenic molecules and induction of fibrolytic enzymes.

Liver fibrosis is known to persist for a long time even after successful pharmacological treatment of hepatitis; therefore, a fibrinolytic therapy to rapidly reverse advanced fibrosis/cirrhosis would be more advisable. This study indicates that, in the experimental model, repeated cycles of hepatic damage have contributed to a significant increase in deposition of collagen fibers mediated by profibrogenic cytokines and that the treatment with the disulfide form of ovothiol A has led to the reversion of this condition. In our model, ovothiol A inhibits GGT activity and affects GSH metabolism. This could be the specific mechanism by which ovothiol negatively regulate redox homeostasis and the activation of key fibrotic markers, TGF-*β*, *α*-SMA, and TIMP1, which finally leads to a significant degradation of the collagen fibers in the ECM (see scheme in Figure 6). To our knowledge, this is the first study to highlight the involvement of the membrane-bound GGT form in the evolution of liver fibrosis, thus pointing to this enzyme as a potential target of therapeutic strategies directed to ameliorate liver fibrosis effects.

Overall, these results suggest that ovothiols can be considered a novel class of sulfur-containing molecules endowed with antifibrotic properties with possible applications as drugs or food supplement for the treatment of chronic inflammation of the liver. This study also highlights the key involvement of sulfur groups in the anti-inflammatory properties of natural products.

## Data Availability

The data used to support the findings of this study are included within the article.

## Abbreviations

ECM: Extracellular matrix
HSCs: Hepatic stellate cells
*α*-SMA: α-Smooth muscle actin
TGF-*β*: Transforming growth factor
TIMP-1: Tissue metalloproteinases inhibitor
GGT: *γ*-Glutamyl transpeptidase
MMPs: Metalloproteinases
AST: Serum aspartate aminotransferase
ALT: Alanine aminotransferase
ALP: Alkaline phosphatase
GAPDH: Glyceraldehyde 3-phosphate dehydrogenase
CCl_4_: Carbon tetrachloride
Col1a1: Fibrillar type collagen 1
NT: Nontreated.
